# Alterations of the Bile Microbiome in Recurrent Common Bile Duct Stone

**DOI:** 10.1155/2020/4637560

**Published:** 2020-09-29

**Authors:** Cheng Ye, Wence Zhou, Hui Zhang, Long Miao, Gen Lv

**Affiliations:** ^1^The First Clinical Medical College, Lanzhou University, Lanzhou, Gansu 730000, China; ^2^General Surgery Department, The First Hospital of Lanzhou University, Lanzhou, Gansu 730000, China

## Abstract

**Objective:**

Common bile duct stone (CBDS) recurrence is associated with bile microbial structure. This study explored the structure of bile microbiome in patients with recurrent CBDS, and its relationship with the recurrence of CBDS.

**Methods:**

Patients with recurrent CBDS (recurrence group) and controls without CBDS (control group) requiring endoscopic retrograde cholangiopancreatography (ERCP) were prospectively included. The control group was noncholelithiasis patients, mainly including benign and malignant biliary stenosis. Bile samples were collected, and bile microbiome structure was analyzed by the 16S rRNA encoding gene (V3–V4).

**Results:**

A total of 27 patients in the recurrence group and 19 patients in the control group were included. The diversity of bile microbiome in the recurrence group was significantly lower than that in the control group (Shannon index: 2.285 vs. 5.612, *P* = 0.001). In terms of bile microbial distribution, patients with recurrent CBDS had significantly higher Proteobacteria (86.72% vs. 64.92%, *P* = 0.037), while Bacteroidetes (3.16% vs. 8.53%, *P* = 0.001) and Actinobacteria (0.29% vs. 6.74%, *P* = 0.001) are significantly lower compared with the control group at the phylum level. At the genus level, the recurrence group was mainly the Escherichia, and there was a variety of more evenly distributed microbiome in the control group, with significant differences between the two groups.

**Conclusion:**

The diversity of bile microbiome in patients with recurrent CBDS is lower. Patients with recurrent CBDS may have bile microbial imbalance, which may be related to the repeated formation of CBDS.

## 1. Introduction

Common bile duct stone (CBDS) is a common and frequently occurring disease of the digestive system. The incidence of gallstones in the United States is about 15%, among which 10%~15% of patients are accompanied by CBDS [1]. The formation of CBDS is the result of a combination of environmental and genetic factors, and the main component is calcium bilirubin. At present, endoscopic retrograde cholangiopancreatography (ERCP) is an important way to treat CBDS [2], but there are often problems with postoperative recurrence [3], and the incidence of multiple recurrences was not uncommon after the first recurrence of CBDS [4].

In the past few years, the relationship between gastrointestinal microbiome and cholelithiasis has been widely studied [5–7]. However, there has been relatively little research on biliary tract microbiology. Currently, some researchers have used 16S rRNA gene-based sequencing to analyze the bile microbiome in patients with gallstones. Both patients with gallstones and nonpathological bile have their own microbiome, and the relative abundance of the two groups in different taxa is significantly different [8]. Studies have shown that all bile microbiome can be detected in the upper digestive tract in CBDS patients, and the bile microbiome was more similar to those of the duodenal fluid samples than to those of the gastric fluid or saliva samples [9]. It is also observed that oral cavity and respiratory tract microbiome are more prevalent in bile samples than intestinal microbiome in CBDS patients [10]. It can be inferred that there are many changes in the bile microbiome in patients with gallstones. Nevertheless, the characteristics of the bile microbiome in patients with recurrent CBDS have not been studied so far.

Thus, we conducted the first study on the characteristics of the bile microbiome in patients with recurrent CBDS and nonbiliary stones, with the aim of obtaining the microbiome profile, function, and activity of bile, which can help us better understand the microbial factors of recurrent CBDS.

## 2. Materials and Methods

### 2.1. Patients and Bile Samples

All patients with recurrent CBDS (recurrence group) and controls (control group) were recruited at the First Hospital of Lanzhou University. The diagnosis of recurrent CBDS was established based on the preoperative imaging evidences by using B-mode ultrasonography, computed tomography (CT) or magnetic resonance cholangiopancreatography (MRCP), and a history of CBDS removal with bile duct surgery or ERCP six months ago, according to most recent research [11, 12].

Exclusion criteria for the recurrence group were residual CBDS that could not be completely removed by previous ERCP or bile duct surgery, previous biliary procedure within the last six months, patient age < 18 years, severe cardiopulmonary complications, and any evidence of secondary CBDS (mainly gallstones or intrahepatic bile duct stones that drain into the bile duct and stay in the bile duct). In order to reduce the impact of geographical location, eating habits, and antibiotic use on the bile microbiome, all the enrolled participants were mixed diet residents in western China and did not receive any antibiotic treatment within 6 months before ERCP [13].

ERCP indication for recurrent CBDS and controls was cholestasis caused by stone incarceration and biliary stenosis, respectively. In controls, CBDS was excluded by preoperative imaging or intraoperative cholangiography. During ERCP, a strictly sterile side-view duodenoscope was used. When the endoscope entered the stomach and duodenum through the oral cavity, the working channel of the endoscope avoided any suction operation, thus keeping it from contamination. After successful biliary intubation, approximately 5 mL bile was extracted by connecting the sterile catheter through the working channel with a sterile syringe before contrast media injection from each patient. The samples were immediately placed into sterile test tubes and stored at -80°C until further processing. This study was conducted according to the 1975 Declaration of Helsinki and was approved by the Ethics Committee of the First Hospital of Lanzhou University.

### 2.2. DNA Extraction and 16S rRNA High-Throughput Sequencing of Bile Samples

DNA was extracted from 1 mL of bile samples according to the previously described method [14]. Two reverse primers (forward: 5′-CCTACGGGNGGCWGCAG-3′, reverse: 5′-GACTACHVGGGTATCTAATCC-3′) were used to amplify the variable regions V3–V4 of the 16S rRNA gene by polymerase chain reaction (PCR). Each sample was amplified by PCR three times, while three parallel amplification products of the same sample were mixed together. Then, each sample was added to an equal volume of Agencourt AMPure XP PCR Purification Beads (Beckman Coulter, USA) to purify the product, followed by Qubit quantification. Finally, the sequencing library quality check and sequencing on an Illumina MiSeq Benchtop Sequencer (Illumina, USA) with MiSeq Reagents Kit v3 (Illumina, USA) were performed.

### 2.3. Data Analysis

The analysis of 16S rRNA high-throughput sequencing data was performed on the platform of QIIME (v1.9.0), which could set the 16S rRNA sequence with a similarity higher than 97% as an operational taxonomic unit (OTU) and perform microbial diversity analysis. Statistical analysis was performed using R 3.6.0 (http://www.rproject.org/) and SPSS v22.0 (IBM, USA). In general, microbial community analyses were carried out mainly using the vegan package. Patient characteristics were presented using descriptive statistics. Continuous variables between two groups were compared using the Mann-Whitney *U* test. Categorical variables were analyzed using the chi-square test or Fisher's exact test. *P* value < 0.05 was considered statistically significant.

## 3. Results

### 3.1. Characteristics of Patients

A total of 46 patients were included for analysis (recurrence group: *n* = 27, control group: *n* = 19). The recurrence group was all patients with recurrent CBDS, accompanied by at least one stone removal history six months ago. It can be found from the image data that all the stones were completely cleared from the previous ERCP or bile duct surgery. The control group consisted of patients without biliary stones, mainly including benign biliary stenosis (*n* = 6), Oddi sphincter dysfunction (*n* = 3), and malignant biliary stenosis (*n* = 8). Benign biliary stenosis was mainly secondary to cholangitis (*n* = 5) and surgical injury (*n* = 1). Malignant biliary stenosis was mainly caused by pancreatic head carcinoma (*n* = 3), bile duct carcinoma (*n* = 4), and ampullary carcinoma (*n* = 1). General characteristics and clinical parameters of the two groups are reported in Table 1. No significant differences were found in terms of age, body mass index (BMI), and main laboratory indicators between the two groups. People that did not undergo ERCP in the recurrence group (25.9%) were included in the previous bile duct surgery group. Compared with the control group, patients in the recurrence group were more likely to have a history of ERCP, cholecystectomy, or bile duct surgery, which may be caused by our inclusion criteria. We speculate that the biliary stable microbial environment is destroyed after surgery, which promotes stone formation and recurrence.

### 3.2. Differences in Bile Microbiome Distribution between the Two Groups

**Figure 1:**
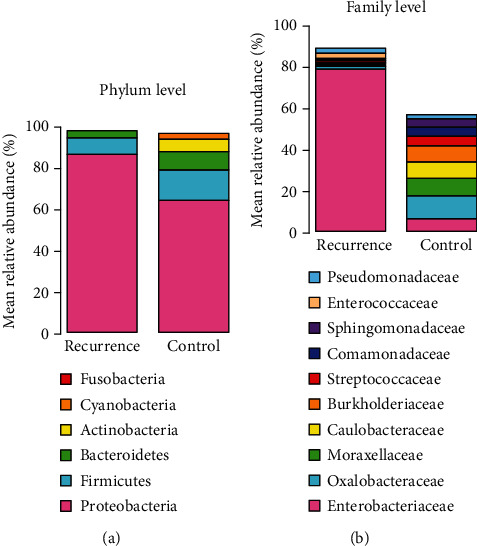
Bar plots representing the mean relative abundance of phylum (a) and family (b) levels in the recurrence group and the control group.

Ductal bile fluid microbiome composition was determined by 16S rRNA high-throughput sequencing technology. The distribution and diversity of bile microbiome were compared between the two groups. Raw reads were averaged 65,404 per sample in the recurrence group and 33,901 in the control group. After quality filtering, an average of 63,501 and 26,354 high-quality 16S rRNA gene sequences was obtained, respectively. We use QIIME and Greengene database (13.8 version) to classify sequences. At the phylum level, Proteobacteria was found to be more abundant in the bile of patients with recurrent CBDS (86.72% vs. 64.92% in the control group, *P* value = 0.037). However, Firmicutes, Bacteroidetes, and Actinobacteria accounted for more than 5% in the control group, and the proportion of Bacteroidetes and Actinobacteria was significantly higher than that in the recurrence group (Mann-Whitney *U* test, *P* = 0.001) (Figure 1(a)). At the family level, Enterobacteriaceae was significantly abundant in the bile samples of the recurrence group (mean relative abundance 79.47%) compared with the control group (mean relative abundance 6.46%). Meanwhile, in the control group, the detection rates of Oxalobacteraceae, Moraxellaceae, Caulobacteraceae, and Burkholderiaceae were higher (10.89%, 8.72%, 8.52%, and 7.58%, respectively) (Figure 1(b)).

**Figure 2:**
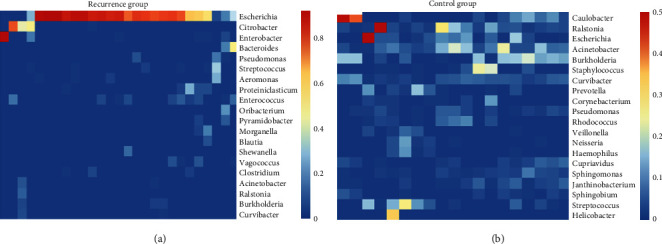
Distribution of core microbiome on the genus level between the recurrence group (a) and the control group (b).

We further explored the genus-level classification of bile microbiome in the two groups. The core microbiome in the recurrence group was dominated by Escherichia, but the richness of the core microbiome decreased rapidly, which was caused by the decreased abundance of genera accounting for more than 1% of the relative richness. However, the control group harboured a variety of evenly distributed core microbiome, including genera from the Alpha (Caulobacter), Beta (Ralstonia and Burkholderia), and Gamma (Acinetobacter and Escherichia) divisions of the Proteobacteria phylum (Figure 2). These results highlight the deviation of patients with recurrent CBDS from the stable core bile microbiome in a healthy state.

### 3.3. Altered Diversity of Bile Microbiome in Recurrent CBDS Patients

**Figure 3:**
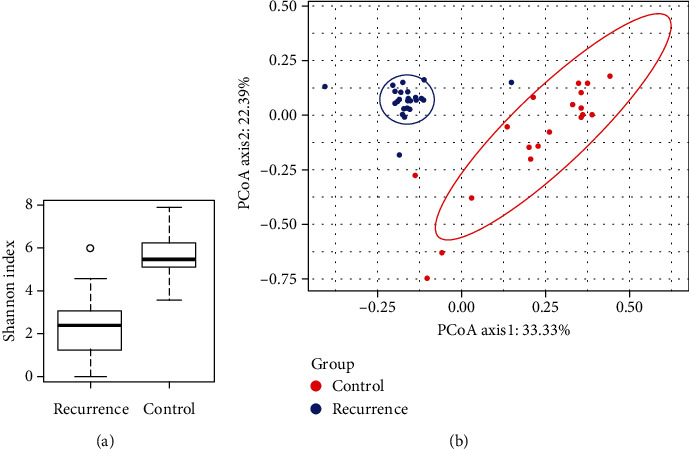
(a) Comparison of Shannon indices in the recurrence group (*n* = 27) and the control (*n* = 19) group. (b) Principal Coordinate Analysis (PCoA) plot of weighted UniFrac distance comparing the bile microbiome in controls and patients with recurrent CBDS.

The within-sample diversity between patients with recurrent CBDS and controls was compared by Alpha diversity. The Shannon, Simpson, Chao1, observed_species, and goods_coverage index for each sample were determined. As can be seen from Table 2 and Figure 3(a), the Shannon and Simpson diversity index was higher in the control group (Mann-Whitney *U* test, *P* value < 0.05). The diversity of bile microbiome in the control group was significantly higher than that in the recurrent CBDS group. However, there was no difference in the Chao1 and observed_species index between the two groups. It shows that there is no obvious difference in the richness of the bacterial community.

The difference of bile microbiome between the recurrence group and the control group could be evaluated by Beta diversity. In view of the weighted UniFrac distance matrix generated by the difference of microbiome between the two groups of bile specimens, we applied Principal Coordinate Analysis (PCoA) to evaluate the similarity between the samples. We found that microbial communities obtained from the bile of patients with recurrent CBDS clustered separately from the control group (Figure 3(b)).

## 4. Discussion

High-throughput sequencing and analysis based on bioinformatics have revolutionized the way we research the human microbiome. With these technologies, we were able to extract genomic information from the gut flora and describe the diversity of that microbial community. Due to the easy availability of noninvasive biological samples, the human fecal microbiome has been extensively studied. Other human microbial sites, however, have been less studied. Recent evidence suggests that natural microbiome exists in the body fluids of various organisms, such as human milk and blood [15, 16].

Bile duct stones are prone to relapse due to a combination of multiple factors [17, 18]. So far, there have been few studies of the human biliary tract microbiome. Only recently, a number of reports have used large-scale sequencing techniques to analyze the bile microbiome of the biliary tract in people who may be involved in the formation of bile duct stones. However, the lack of bile samples for recurrent CBDS hinders the generation of physiologically relevant results and makes it difficult to establish a link between the microbiome and the recurrence of bile duct stones. Liang et al. [19] found that the bile duct microenvironment of patients with sphincter of Oddi laxity had a more serious bacterial infection and stronger lithogenicity by comparing the bile microbiome of CBDS patients with and without sphincter of Oddi laxity. Among the microbiome of the infected bile duct, Proteobacteria and Firmicutes were the most widespread phylotypes, especially Enterobacteriaceae. Researchers have also studied the bacterial communities of the biliary tract, duodenum, stomach, and oral cavity from six gallstone patients by 16S rRNA amplicon sequencing and found that all observed bile microbiome were detectable in the upper digestive tract. Compared with other regions, the bile microbiome had a comparatively higher similarity with the duodenal microbiome, but with a reduced diversity [9]. Shen et al. [10] found similar results when they observed a reduction in the microbial diversity of bile samples in patients with gallstones compared to healthy fecal samples. Our results indicate that bile samples from patients with recurrent CBDS also have reduced microbial diversity compared to nonbiliary duct stone patients. It can be seen that the bile microbiome of patients with CBDS has deviated from a normal stable state, and the recurrence is closely related to the microbial environment of the biliary tract.

To date, microbial populations in the bile ducts of patients with recurrent choledocholithiasis have not been studied. Previous studies have demonstrated the characteristics of the bile microbiome in CBDS patients [19]. This leads us to think whether the recurrence of CBDS is related to the bile microbiome. Therefore, in our study, we have taken another step in trying to represent the bile microbiome of patients with recurrent CBDS. PCoA analysis based on the weighted UniFrac distance divided the bile microbiome of the two study groups into different groups, showing their respective aggregation states. We observed a decrease in the average biodiversity of the bile microbiome in patients with recurrent CBDS. From an ecological perspective, the drop in diversity can erode the ecosystem resilience of a naturally functioning ecosystem, increasing the likelihood of critical ecosystem degradation [20]. In addition, we observed a significant increase in Proteobacteria in the bile of recurrent CBDS at the phylum level. The dysbiotic expansion of Proteobacteria is usually associated with increased epithelial oxygen availability and is a potential diagnostic microbial signature of epithelial dysfunction [21]. The increasing abundance of Proteobacteria is a potential diagnostic signal for dysbiosis and disease risk [22]. Therefore, we can speculate that patients with recurrent CBDS may have biliary dysbiosis. At the genus level, Escherichia is more abundant in the biliary tract of patients with recurrent CBDS from the microbiological heat maps of the two groups. As mentioned earlier, E. coli is common in the bile of cholelithiasis patients [23, 24], and about 70% of gut bacterial OTUs from gallstone patients were detectable in the biliary tract [25]. The unusual existence of virulence gene combinations in Escherichia and their resistance to deoxycholate sodium and multiple classes of antibiotics could be considered as possible causes of their persistence in the biliary microenvironment [26]. In addition, duodenal-biliary reflux is correlated with CBDS recurrence in patients who had previously undergone ERCP [27]. The majority of patients with recurrent CBDS in this study had experienced at least one ERCP, which caused the laxity of the Oddi sphincter and invasion of the intestinal microbiome into the biliary tract, suggesting the role of Escherichia in stone recurrence.

Our study has several limitations. Firstly, our cohort size was small, and our microbiome analysis was limited to single-center cohorts of the same race and diet in western China. A multicenter study cohort should be included in the future to assess the robustness of observed changes in bile microbiome. Secondly, due to the low abundance of bacterial DNA in our bile samples, more reliable metagenomic shotgun sequencing cannot be performed, so the level and diversity of bile microbial species were analyzed by 16S rRNA high-throughput sequencing. Thirdly, because ERCP is an operation related to the health risks of the examinee, bile samples cannot be obtained from healthy volunteers for ethical reasons. Therefore, we selected patients with nonbiliary stones as the control group, such as benign biliary stenosis and bile duct cancer. We cannot confirm whether these nonbiliary duct stone diseases have an impact on the biliary microenvironment. Fourthly, In order to reduce the interference of antibiotics, we choose patients who have not used antibiotics for 6 months before ERCP to minimize the interference of antibiotics on the bile microbiome. But the antibiotic effect cannot be completely ruled out.

## 5. Conclusions

Even taking these limitations into account, our results lay the foundation for future larger-scale studies of the relationship between bile microbiome and human health. This is the first study of the bile microbiome in patients with recurrent choledocholithiasis and has established a link between the bile microbiome and the recurrence of bile duct stones. These findings may provide promising strategies for finding novel disease-related biomarkers that can help prevent the recurrence of stones. More research on the role of microbiome in the pathogenesis of bile duct stones is needed to explore the microbial ecology of the biliary tract in the future.

## Figures and Tables

**Table 1:** Baseline characteristics of patients in the recurrence group and the control group.

Characteristic	Recurrence group	Control group	*P* value
Patients, *n*	27	19	NA
Male, *n* (%)	17 (63%)	11 (57.9%)	0.767
Age (years)	62 (34-90)	53 (14-78)	0.010
BMI (kg/m^2^)	24.6 (18.9-33.7)	23.4 (19.1-32.3)	0.065
TBIL (*μ*mol/L)	34.1 (9.2-183.6)	24.1 (11.9-443.2)	0.260
AST (U/L)	33.2 (10.2-388.7)	28.1 (15.3-295.7)	0.538
ALT (U/L)	71.5 (5.3-641.1)	74.7 (11.2-308.6)	0.378
ALP (U/L)	157.1 (46.5-881.9)	81.4 (50.2-896.5)	0.631
GGT (U/L)	168.6 (14.7-1054.2)	41.9 (13.8-791)	0.259
WBC (10^9^/L)	7.7 (1.6-24.8)	5.7 (2.8-10.2)	0.199
Previous ERCP, *n* (%)	17 (74.1%)	2 (10.5%)	0.001
Previous EST, *n* (%)	12	0	<0.001
Previous cholecystectomy, *n* (%)	22 (81.5%)	2 (10.5%)	<0.001
Previous bile duct surgery, *n* (%)	13 (48.1%)	0	<0.001
Mean recurrence time (year)	2 (0.5-20)	NA	NA

BMI: body mass index; TBIL: total bilirubin; AST: aspartate aminotransferase; ALT: alanine aminotransferase; ALP: alkaline phosphatase; GGT: gamma-glutamyl transpeptidase; WBC: white blood cell; ERCP: endoscopic retrograde cholangiopancreatography; EST: endoscopic sphincterotomy; NA: not available.

**Table 2:** Alpha diversity analysis of biliary microbiota in the recurrence group and the control group.

Alpha diversity index	Recurrence group	Control group	*P* value
Shannon	2.285	5.612	0.001
Simpson	0.537	0.929	0.001
Chao1	485.778	466.849	0.600
Observed_species	366.444	359.789	0.482
Goods_coverage	0.998	0.991	0.013

## Data Availability

The data used to support the findings of this study are available from the corresponding author upon request.
